# 

**Published:** 1894-08

**Authors:** 


### CONTENTS.

ORIGINAL COMMUNICATIONS—

Nausea. By James B. Baird, M.D., Atlanta, Ga ..... 321
The Pathology and Treatment of Gonorrhea. By Charles M. Blackford, Jr., M.D., Lynchburg, Va ..... 325
The Treatment of Syphilis by Hypodermatic Injections, with Special Reference to the Salicylate of Mercury. By L. Amster, M.D., Atlanta, Ga ..... 331
The Therapeutics of Infancy and Childhood. By N. G. Gewinner, M.D., Macon, Ga. 337

SOCIETY REPORTS—

Macon Medical Society ..... 344

Clinical Society of Maryland ..... 346

CORRESPONDENCE—

A Few Surgical Observations ..... 349

EDITORIAL—

The Legislative Control of Medical Practice ..... 351
Medicine as She is Taught ..... 354
Dr. William Thomas Coggin ..... 356
The Atlanta Obstetrical Society ..... 357

SELECTIONS AND ABSTRACTS—

Surgery ..... 359
General Medicine and Therapeutics ..... 367
Practical Chemistry and Pharmacy ..... 375

MEDICAL ITEMS ..... 378
BOOK REVIEWS ..... 380
ADVERTISERS' INDEX TO ADVERTISEMENTS ..... xii
CLASSIFIED INDEX TO ADVERTISEMENTS ..... xxxvii
ITEMS OF INTEREST ..... x, xi

